# Isha yoga practices, vegan diet, and participation in Samyama meditation retreat: impact on the gut microbiome & metabolome – a non-randomized trial

**DOI:** 10.1186/s12906-023-03935-8

**Published:** 2023-04-05

**Authors:** Maitreyi Raman, Ramana Vishnubhotla, Hena R. Ramay, Maria C. B. Gonçalves, Andrea S. Shin, Dhanashri Pawale, Balachundhar Subramaniam, Senthilkumar Sadhasivam

**Affiliations:** 1 Cumming School of Medicine, University of Calgary, Calgary, AB Canada; 2 Department of Radiology and Imaging Sciences, Indiana University School of Medicine, Indianapolis, IN USA; 3 International Microbiome Centre, Cumming School of Medicine, University of Calgary, Calgary, AB Canada; 4 Department of Anesthesia, Critical Care and Pain Medicine, Sadhguru Center for a Conscious Planet, Beth Israel Deaconess Medical Center, Boston, MA USA; 5 Division of Gastroenterology and Hepatology, Indiana University School of Medicine, Indianapolis, IN USA; 6 Department of Anesthesiology and Perioperative Medicine, University of Pittsburgh School of Medicine, Pittsburgh, PA USA

**Keywords:** Meditation, Yoga, Vegan diet, Microbiome, Brain-gut axis

## Abstract

**Background:**

Growing evidence suggests a role for gut bacteria and their metabolites in host-signaling responses along the gut-brain axis which may impact mental health. Meditation is increasingly utilized to combat stress, anxiety, and depression symptoms. However, its impact on the microbiome remains unclear. This study observes the effects of preparation and participation in an advanced meditation program (Samyama) implemented with a vegan diet including 50% raw foods, on gut microbiome and metabolites profiles.

**Methods:**

There were 288 subjects for this study. Stool samples were collected at 3-time points for meditators and household controls. Meditators prepared for 2 months for the Samyama, incorporating daily yoga and meditation practices with a vegan diet including 50% raw foods. Subjects were requested to submit stool samples for 3 time points – 2 months before Samyama (T1), right before Samyama (T2), and 3 months following Samyama (T3). 16 s rRNA sequencing was used to study participants' microbiome. Alpha and beta diversities along with short-chain fatty acid (SCFA) were assessed. Metabolomics were performed on a mass spectrometer coupled to a UHLPC system and analyzed by El-MAVEN software.

**Results:**

Alpha diversity showed no significant differences between meditators and controls, while beta diversity showed significant changes (padj = 0.001) after Samyama in meditators’ microbiota composition. After the preparation phase, changes in branched short-chain fatty acids, higher levels of iso-valerate (padj = 0.02) and iso-buytrate (padj = 0.019) were observed at T2 in meditators. Other metabolites were also observed to have changed in meditators at timepoint T2.

**Conclusion:**

This study examined the impact of an advanced meditation program combined with a vegan diet on the gut microbiome. There was an increase in beneficial bacteria even three months after the completion of the Samyama program. Further study is warranted to validate current observations and investigate the significance and mechanisms of action related to diet, meditation, and microbial composition and function, on psychological processes, including mood.

**Trial registration:**

Registration number: NCT04366544; Registered on 29/04/2020.

## Background

Understanding the influence of the gut microbiota on nervous system function is gaining increasing interest from the scientific community [1–3]. Ailments linked to the gut-brain axis include inflammatory bowel diseases, irritable bowel syndrome [4, 5], Parkinson’s diseases, anxiety, and depression [6–8]. Clarifying the impact of psychological interventions on the microbiota and vice versa could enhance the effectiveness and scope of therapeutic approaches for psychological, neurological, and digestive diseases.

The human gut microbiota influences emotional and psychological states in a bidirectional way. Recent evidence points to the microbiome as part of a neuro-immune-endocrine matrix [9, 10]. Bacteria native to the gut can activate neural pathways that participate in anxiety and depression [6, 11]. These include the hypothalamus–pituitary–adrenal (HPA) axis, a neuroendocrine system linked to stress response [12–15], which regulates the gut-brain signaling, and may influence stress related diseases such as anxiety and depression [8]. The reverse is also true as stress can alter the microbiota leading to increasing inflammation and lowering the anti-inflammatory and anti-tumor effects of a healthy microbiome [16].

Over the past decade, meditation has become an increasingly popular method to address symptoms related to stress, anxiety, and depression. Meditation effectively reduces symptoms associated with anxiety and depression [17–19]. Considering the potential impact of meditation on the stress response, meditation may additionally impact the gut microbiota [11, 20] and modulate the gut-brain axis [21]. Meditators demonstrate differences in the gut microbiota compared to non-meditators, which is characterized by enrichment of beneficial bacterial genera such as *Bifidobacterium*, *Roseburia*, and *Subdoligranulum* [11, 16]. We have previously reported that advanced meditation improves symptoms associated with anxiety and depression [22], results in positive psychological metrics [22, 23], reduces the expression of blood biomarker of inflammation [22], and increases the expression of endocannabinoids and brain-derived neurotrophic factor (BDNF) [23, 24]. Additionally, we have observed alterations following Samyama in functional brain connectivity between the salience and default mode networks in meditators [25], along with increased gene expression of immune signaling molecules that are relevant for diseases such as COVID-19 and multiple sclerosis [26]. We have also seen that there are significant changes in the lipidomics profile after Samyama [27]. In this study, we aimed to assess the impact of Samyama, an advanced meditation program that includes a preparatory phase of 60-day vegan diet requirement on the gut microbiome. We will measure outcomes for 1) microbiota composition and 2) metabolite composition.

## Materials and methods

### Ethics statement

**Fig. 1:**
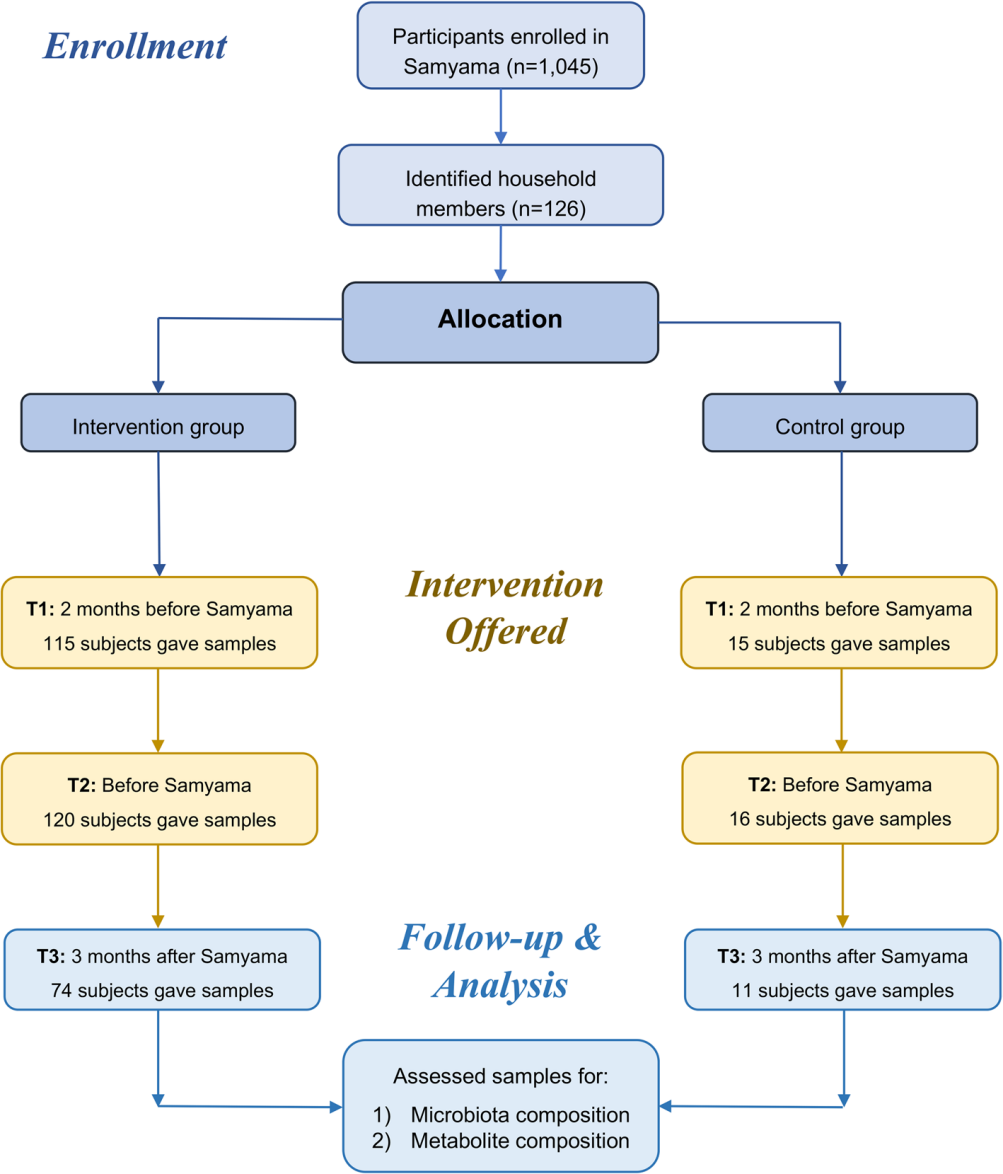
CONSORT diagram

This study was conducted in accordance with the Declaration of Helsinki [28]. It was reviewed and approved by the Indiana University School of Medicine Internal Review Board (IRB) (#1,801,728,792). Subjects provided electronic informed consent for this study after completing initial electronic surveys. This study adheres to CONSORT guidelines [29] (Fig. 1).

### Subject recruitment

The study was registered in ClinicalTrials.gov (reg #: NCT04366544) on 29/04/2020. Subjects for this non-randomized parallel controlled study were recruited from the same participant pool as a previously published study [22]. The Isha Institute of Inner Sciences (McMinnville, TN) provided a list of registrants for the April 2018 Samyama Program [30]. Invitation letters with study information were sent electronically to all registrants 2–3 months before the program. Study participants were recruited from the United States. Spouses or other household members were recruited as non-randomized control subjects. Subject recruitment included a cohort of meditators and cohort of spouses who were not enrolled in the program. There were 759 subjects enrolled in the studies, of which 288 (265 Samyama participants, 23 household controls) participated in this study. Study eligibility criteria included: Advanced meditation program participants at least 18 years of age. Exclusion criteria: Inability to read or comprehend the consent form; subjects with medical conditions in which a blood draw would be contraindicated (e.g., severe anemia); active use of marijuana, opioids, or related drugs; use of antibiotics or probiotic/prebiotic supplements within 60 days of enrollment; participants living outside of the United States. Spouses who actively participated in meditation were also excluded from the spousal control group. Group details can be found in Table 1.

**Table 1:** Demographic data

	Meditators (*N* = 265)	Controls *(N* = *23)*
**Age *(years)***	40.7 ± 10.9	42 ± 1.41
**Gender**		
Female	139	10
Male	126	13
**Weight *(lbs)***	141 ± 27.6	165 ± 26.8
**BMI**	22.9 ± 3.83	26.2 ± 3.24
**Timepoints**		
T1	115	15
T2	120	16
T3	74	11

### Samyama program – preparatory process

#### Dietary requirements

As part of the Samyama preparatory process (60 days before the program), Samyama participants (meditators) followed a vegan diet with at least 50% raw foods consumed. Additionally, they were requested to avoid dietary intake of garlic, onion, chili, eggplant, *asafoetida*, coffee, and tea. Finally, alcohol, cigarettes, stimulants, and illicit drugs were discouraged. Controls were not required to follow any dietary requirements.

#### Meditation practice requirements

Samyama participants (meditators) were required to take several prerequisite meditation programs before enrolling in Samyama. These included Inner Engineering [31], Bhava Spandana [32], Shoonya [33], and Yogasanas [34]. They were asked to perform multiple practices (learned in prerequisite programs) daily for the 60-day preparation period. These include kriya yoga practices (Shakti Chalana Kriya and Shambhavi Mahamudra Kriya), hata yoga (Surya Kriya and Yogasanas), Shoonya meditation twice a day, Sukha Kriya and Arda Siddhasana for at least 1 h per day. Kriya yoga practices are combinations of posture, breath, and sound. Hata yoga practices consist of physical postures. Shoonya meditation is a process of conscious non-doing. Sukha Kriya consists of alternate nostril breathing, which leads to regulation of breath. Ardha Siddhasana is a posture in which one sits cross-legged with the heel of the left foot placed at the perineum. Controls did not have any pre-program practice requirements.

### Samyama program – retreat

During the program, meditators were to remain silent for the entire 8-day duration of the program. They took part in all-day meditation sessions with intermittent breaks. The program hall was closed to external influences. No specific instructions or programs were given to the controls. Upon completing the Samyama program, there were no further restrictions on meditators. Meditators were able to return to their previous lifestyle.

### Stool sampling

Subjects were requested to submit stool samples for 3 time points – 2 months before Samyama (T1), immediately before Samyama (T2), and 3 months following Samyama (T3). Stool samples were collected from meditators and control subjects in sterile fecal collection containers. Upon receiving samples from participants, they were packaged with cold packs and shipped overnight. The samples were then stored at -80 ℃ until samples were analyzed.

### Metabolomics

Metabolomics were performed as described previously [35, 36]. Briefly, metabolomics runs were performed on a Q Exactive™ HF Hybrid Quadrupole-Orbitrap™ Mass Spectrometer (Thermo-Fisher) coupled to a Vanquish™ UHPLC System (Thermo-Fisher). Chromatographic separation was achieved on a Syncronis HILIC UHPLC column (2.1 mm x 100 mm × 1.7μm, Thermo-Fisher) using a binary solvent system at a flow rate of 600μL/min. Solvent A, 20 mM ammonium formate pH 3.0 in mass spectrometry grade H_2_O; Solvent B, mass spectrometry grade acetonitrile with 0.1% formic acid (%v/v). A sample injection volume of 2μL was used. The mass spectrometer was run in negative full scan mode at a resolution of 240,000 scanning from 50-750 m/z.

Metabolite data was analyzed by El-MAVEN software package and identified by matching observed m/z signals (± 10 ppm) and chromatographic retention times to those observed from commercial metabolite standards (Sigma-Aldrich) [37, 38]. Next, metabolites were quantified by comparison to an eight-point quantification curve of metabolite standards.

### Microbiota composition and data analysis

We used 16 s rRNA sequencing to study the participants' microbiome over time. Sequencing data from MiSeq was de-multiplexed and converted to FASTQ format using Illumina's bcl2fastq (RRID:SCR_015058) software. CutAdapt was used for initial quality trimming [39]. DADA2 v1.10.1 pipeline was used to generate and ASV (amplicon sequence variant) table. Taxonomy was assigned to the representative sequences using the idTaxa classifier [40] from DECIPHER R package using SILVAv.132 database [41]. Downstream analysis was done in R using phyloseq 1.30.0 [42]. Alpha diversity, summarizing the structure of a community with respect to its richness and evenness in a sample [43–45], was measured by calculating the Shannon and Chao1 index. Beta diversity, used to assess changes in microbial community across different groups, was visualized using principal coordinate analysis (PCoA) on the Bray–Curtis dissimilarity matrix, and changes in the bacterial community were assessed statistically using PERMANOVA. The short-chain fatty acid (SCFA) data was converted to proportion by dividing each SCFA by total SCFA concentration in a sample. If the data was normally distributed, parametric one-way analysis of variance (ANOVA) with Tukey's post-hoc tests was used. All measurements were normalized using autoscaling for metabolomics data, and then t-test was performed with adjustment for multiple testing.

## Results

### Demographic data

There were 265 meditators enrolled in this study, including 139 females and 126 males with an average age of 40.7 ± 10.9 years, and 23 house-hold controls including 10 females and 13 males with an average age of 42 ± 1.41. Participants’ demographic data is summarized in Table 1.

### Microbial diversity

**Fig. 2:**
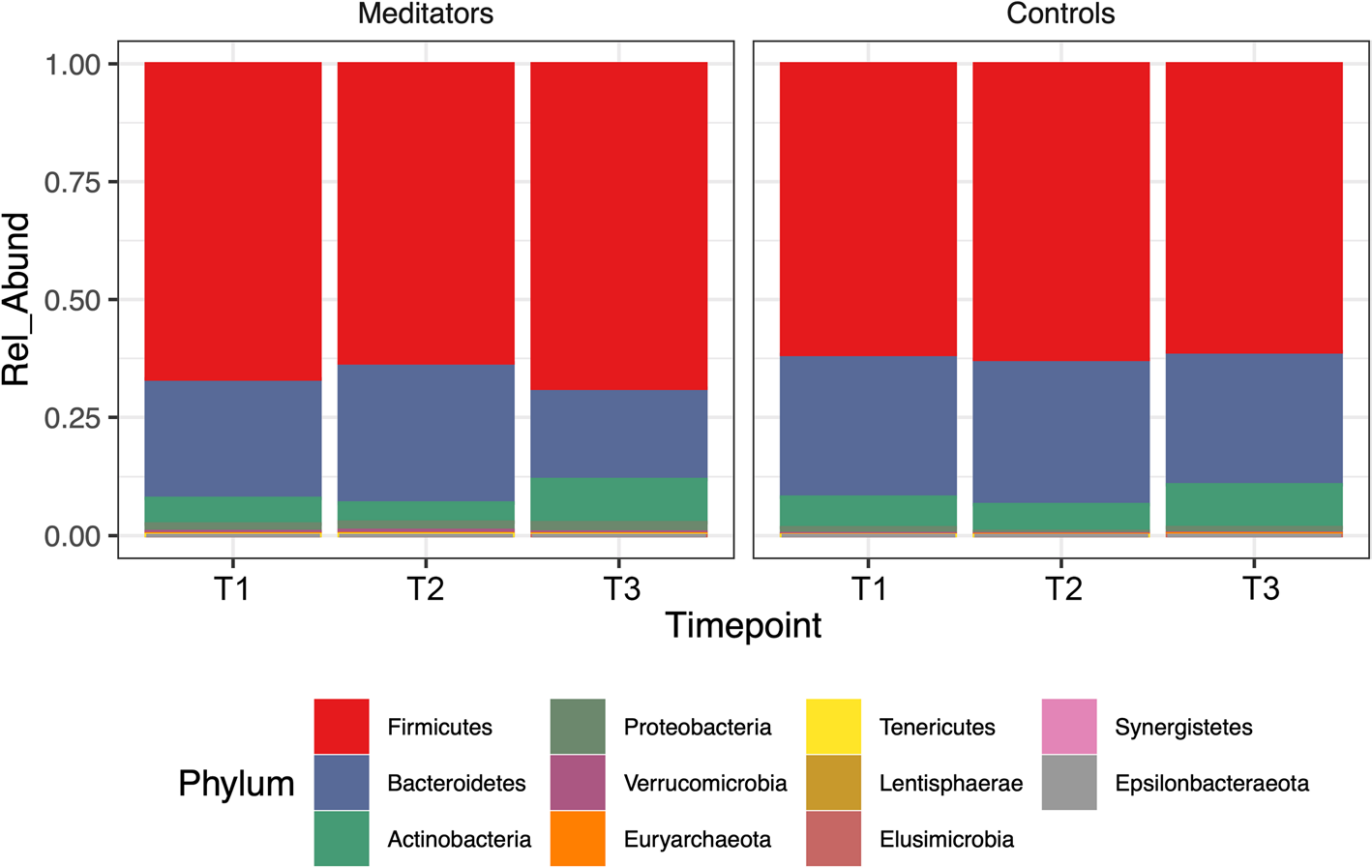
Charts show phylum level relative abundance profile for meditators and controls at 3-time points

**Fig. 3:**
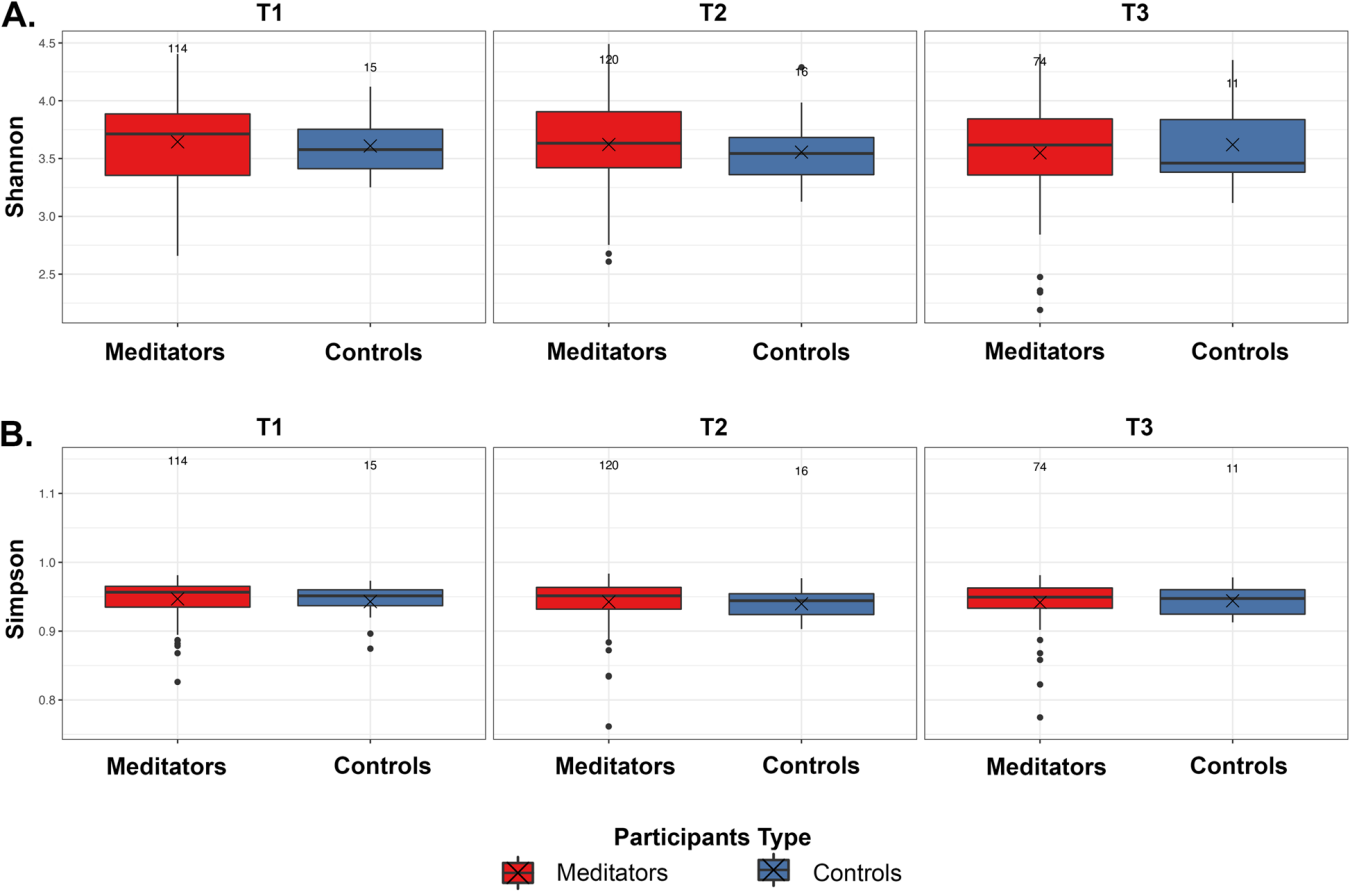
Alpha diversity plots **A** Boxplots of Shannon index for participants over time. **B** Boxplots of Simpson index for participants over time

**Fig. 4:**
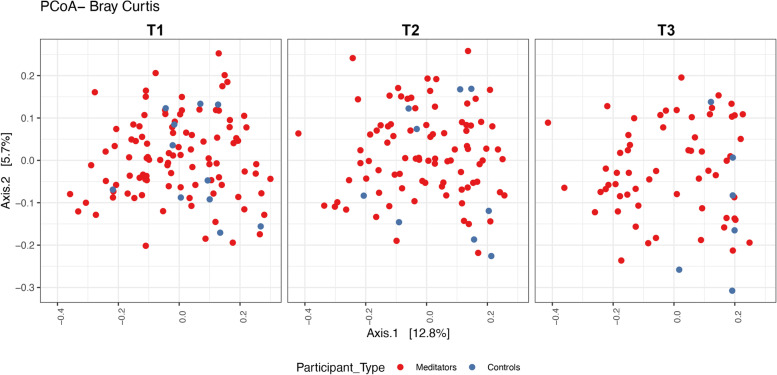
Beta diversity plots. PCoA was used to visualize the Bray–Curtis similarity for all participants over time

Figure 2 shows the phylum level relative abundance profile of all participants over time (Table 2). With these data we looked at alpha diversity to assess sample specific microbial composition and beta diversity to study the microbial community structure. Figure 3 shows two different alpha diversity measures, Shannon and Simpson, which resulted in no differences across or within meditator and control groups at all three time points. However, analysis of fecal microbiota beta-diversity demonstrated statistical differences between time points T2 and T3 within meditators (padj = 0.001) (Fig. 4).

**Table 2:** Phylum level relative abundance values for timepoints and types

Taxa	Meditators_T1	Controls_T1	Meditators_T2	Controls_T2	Meditators_T3	Controls_T3
*Bacteroidetes*	2.60E-01	3.00E-01	3.00E-01	3.00E-01	2.00E-01	2.40E-01
*Firmicutes*	6.60E-01	6.10E-01	6.30E-01	6.40E-01	6.80E-01	6.50E-01
*Actinobacteria*	5.70E-02	6.70E-02	4.20E-02	5.30E-02	9.60E-02	1.00E-01
*Proteobacteria*	1.70E-02	1.30E-02	1.90E-02	4.30E-03	2.20E-02	8.30E-03
*Verrucomicrobia*	5.10E-03	2.60E-03	7.50E-03	2.10E-03	4.10E-03	1.90E-03
*Euryarchaeota*	2.20E-03	1.00E-03	2.00E-03	2.80E-03	2.40E-03	2.60E-03
*Lentisphaerae*	7.20E-04	4.80E-04	7.50E-04	4.50E-05	1.90E-04	0.00E + 00
*Tenericutes*	1.90E-04	0.00E + 00	5.70E-04	0.00E + 00	1.80E-04	0.00E + 00
*Elusimicrobia*	5.10E-06	0.00E + 00	3.00E-06	0.00E + 00	1.90E-04	9.20E-04
*Synergistetes*	9.40E-06	7.30E-06	8.70E-06	0.00E + 00	0.00E + 00	0.00E + 00
*Epsilonbacteraeota*	2.70E-07	0.00E + 00	4.30E-06	0.00E + 00	0.00E + 00	0.00E + 00

### Differential abundance analysis

**Fig. 5:**
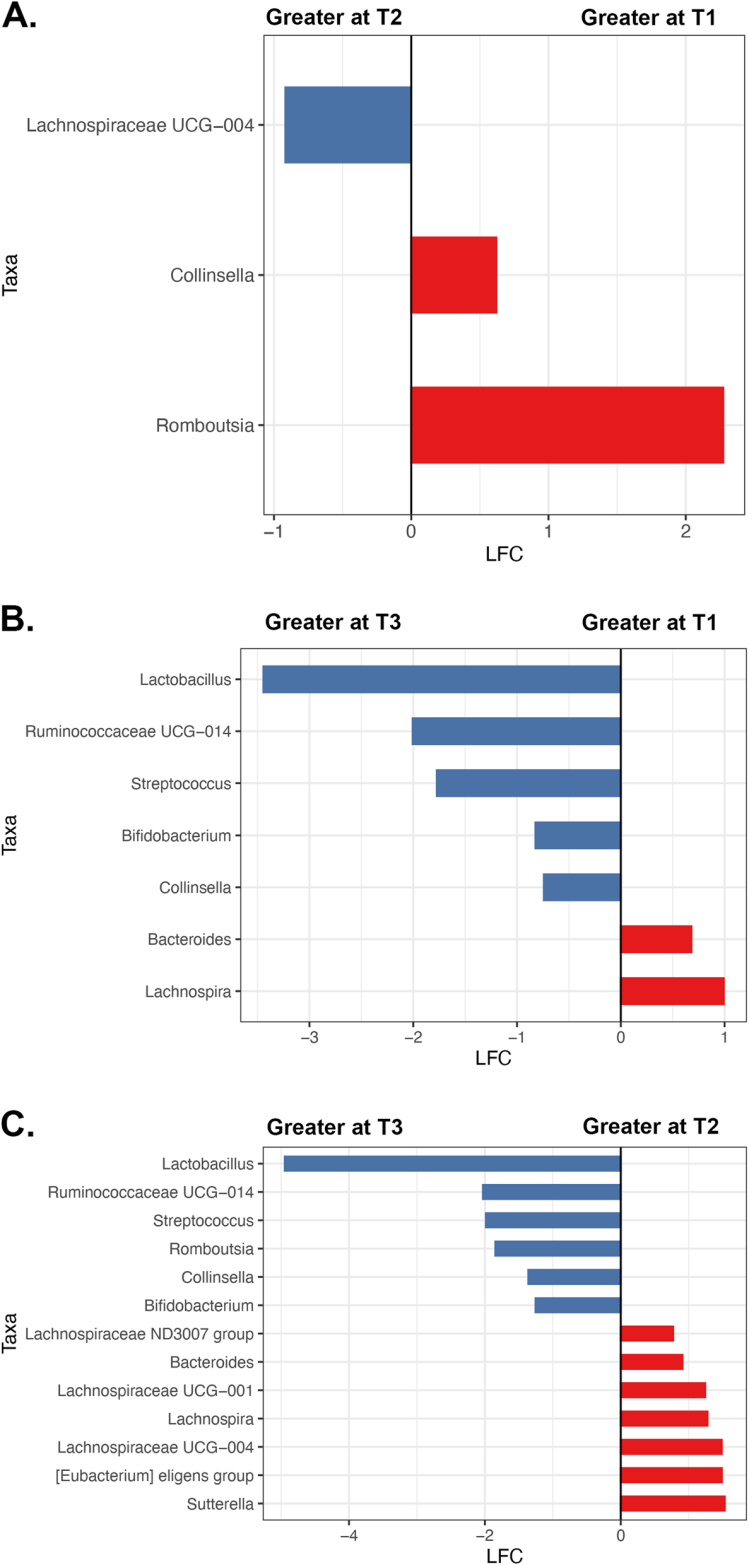
Taxa enrichment genus level results for meditators. **A** Baseline vs. Before Meditation (T1 vs. T2), **B** Baseline vs. After Meditation (T1 vs. T3), **C** Before vs. After Meditation (T2 vs. T3)

To pinpoint the differences in the microbiota (genus level) we conducted differential abundance analysis over time in participants. We did not find any significantly different taxa in control samples over time (data not shown); hence we focused here on meditators only. Among meditators, *Lachnospiraceae UCG-004* population increased (LFC = 0.91, padj = 0.0211) while *Romboutsia* (LFC = -2.2, padj = 5.1e-6) and *Collinsella* (LFC = -0.61, padj = 0.078) populations decreased at T2 from T1 (Fig. 5A). At timepoint T3 compared to T1, *Lactobacillus* (LFC = -3.4, padj = 0.02), *Bifidobacterium* (LFC = 0.82, padj = 0.003), *Ruminococcaceae UCG-014* (LFC = 2.0, padj = 0.001) and *Streptococcus* (LF = 1.72, padj = 9.5e-6) populations increased while Bacteroides (LFC = -0.68,padj = 0.008) and *Lachnospira* (LFC = -0.92,padj = 0.01) populations decreased (Fig. 5B). The greatest changes were observed between T2 and T3 where *Bacteroides* (LFC = -0.9,padj = 5.1exp6), *Lachnospiraceae UCG-004* (LFC = -1.48,padj = 4.6exp-5)*, Lachnospiraceae UCG-001* (LFC = -1.24,padj = 0.03)*, Lachnospira* (LFC = 1.28,padj = 2.3exp-4) *and* [*Eubacterium] eligens group* (LFC = -1.48,padj = 9.2exp-5) populations decreased in T3, while *Lactobacillus* (LFC = 4.9,padj = 4.7exp-7), *Ruminococcaceae* UCG-014 (LFC = 2.03,padj = 0.017), *Romboustia* (LFC = 1.84,padj = 0.0018), *Collinsella* (LFC = 1.36,padj = 1.67exp-10) and *Bifidobacterium* (LFC = 1.25,padj3.67e-7 =) increased in T3 (Fig. 5C). Overall, by timepoint T3, Lactobacillus, Bifidobacterium, Ruminococcaceae, Streptococcus, and Collinsella populations were increased compared to both previous timepoints.

### Short-chain fatty acid levels

**Fig. 6:**
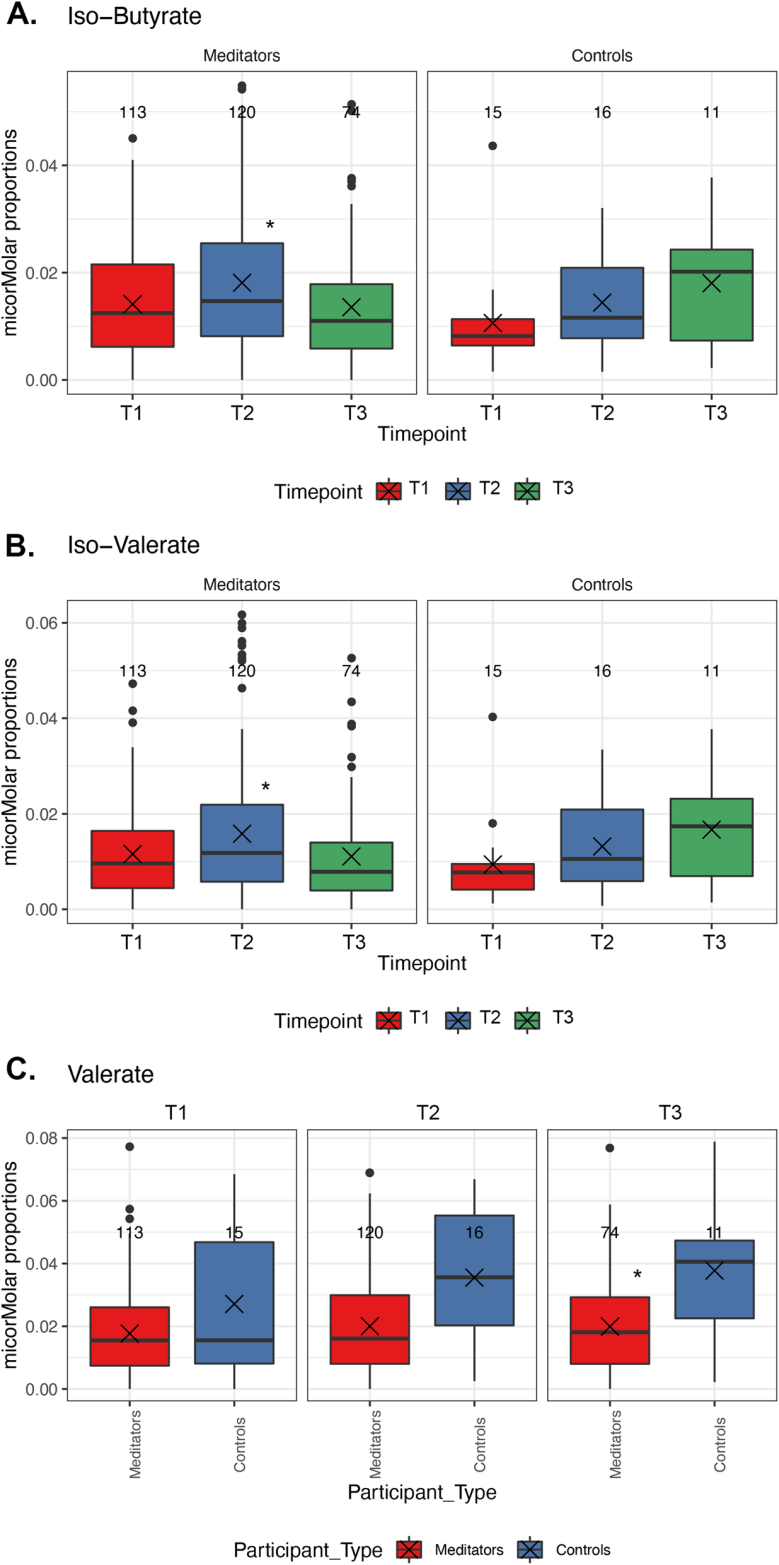
SCFA showed a significant difference. **A** Iso Butyrate and **B** Iso-Valerate showed significant differences within meditators at T2 vs. T1 and T2 vs. T3. **C** Valerate showed significant differences in meditators vs. controls in T2. Significance is marked with an asterisk (*)

We further investigated short chain fatty acid (SCFA) levels in participants over time. While we did not find differences over time in the proportions of SCFAs ascribed to carbohydrate metabolism (butyrate, acetate and propionate), we found that within meditators, branched SCFAs including iso-butyrate (Fig. 6A) and iso-valerate (Fig. 6B) levels were greater at T2 compared to T1 (padj = 0.019) and T3 (padj = 0.02). We also observed a lower proportion of valerate in meditators compared to controls at timepoint T3 (padj = 0.012) (Fig. 6C). No changes were seen within the controls.

### Other metabolites’ profiling

**Fig. 7:**
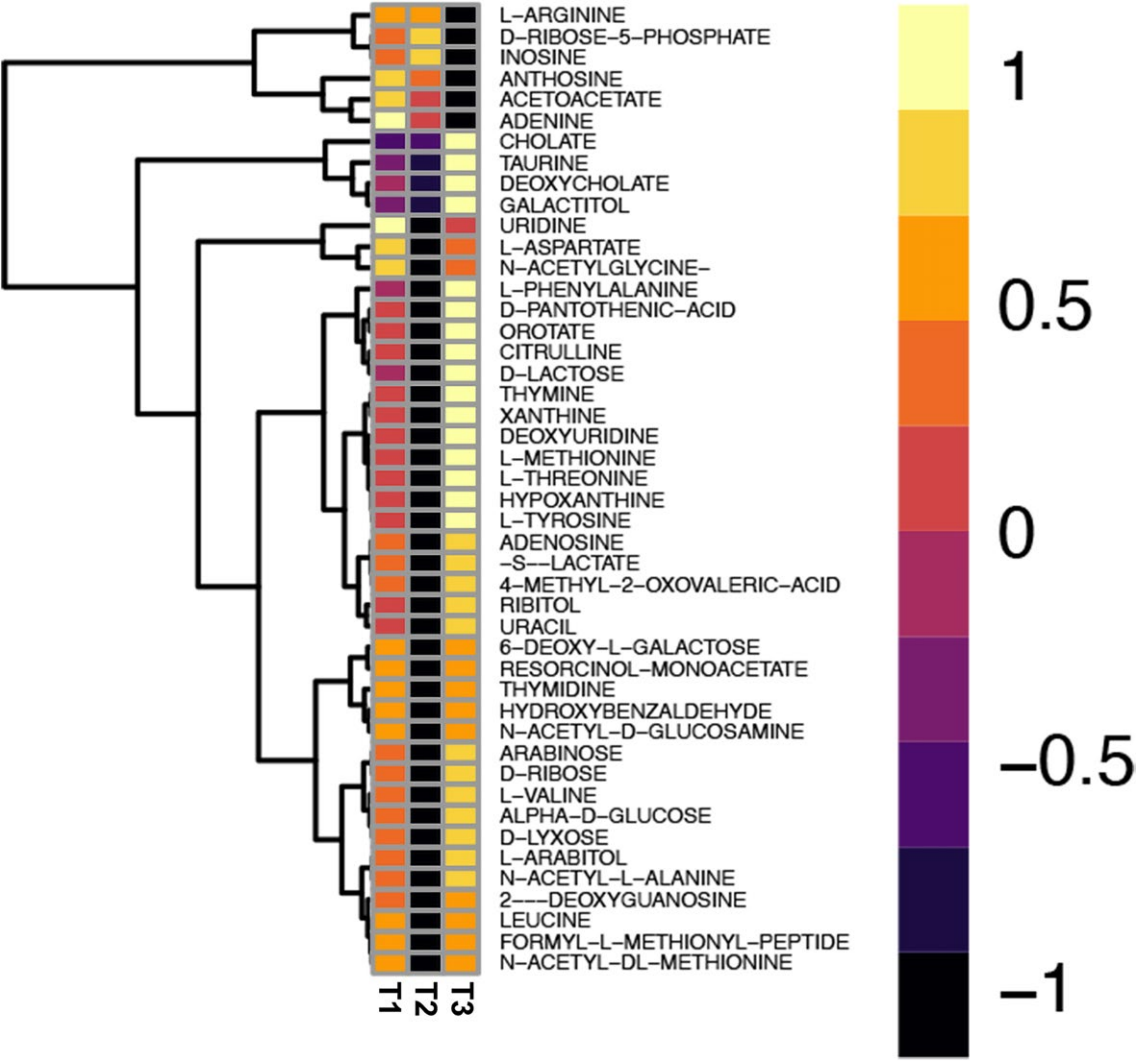
Metabolomics intensity in log2 units. Heatmap of metabolites that have significantly changed across two timepoints. Most metabolites that show significant change are decreased in T2

We also explored the changes in other microbial-derived metabolites over time in the participants (untargeted metabolomics analysis). While we did not find any metabolites to be significantly different in control participants over time, we found 46 metabolites to be significantly different in meditators overtime points. Most of these metabolites decreased in T2 and subsequently increased at T3 (Fig. 7).

## Discussion

This non-randomized controlled study is among the first and largest to explore the gut microbiome and metabolome after an advanced meditation program delivered with a vegan diet. We demonstrated associations of beta diversity with time points T2 and T3 in the meditation group, suggesting compositional changes in the microbiota arising from either meditation or possibly returning to habitual diet from a vegan diet. However, measures of alpha diversity in the gut microbial communities were not different between groups at any time points. Additionally, we tested for sex differences in alpha and beta diversities (at each timepoint) but did not find any significant differences. This may be a consequence of a healthy, unselected participant group. We did not find any taxa to be differentially abundant between meditators and controls at any timepoint; however, an altered abundance of several genera was observed in meditators with changes of the greatest magnitude observed at T3 compared to T2. Furthermore, we demonstrated several changes in fecal metabolites over time.

*Lachnospiraceae* increased in abundance at T2 compared to T1, during the preparation phase. *Lachnospiraceae* has been linked to SCFAs production [46, 47], which was observed in our study by increased levels of iso-butyrate and iso-valerate in meditators during this time period. *Lachnospiraceae* can beneficially and adversely affect disease progression for a variety of ailments, including metabolic disorders, diabetes, inflammatory bowel disease, and depression [47]. Some taxa of *Lachnospiraceae* were shown to be positively correlated to a diet rich in unprocessed/raw foods, while others were correlated to higher intake of processed foods and meats [48]. We tested each SCFA for sex differences but except for iso-butyrate in T2 (*p*value = 0.02), we did not see any significant differences in the sexes. Another study showed an increase in *Lachnospiraceae* population after implementing a vegetarian diet [49]. This study validates previous reports that vegan diet increases *Lachnospiraceae* microbe in our study population of meditators practicing predominantly vegan diet.

We demonstrated increased abundance of *Lactobacillus* and *Bifidobacterium* at T3 compared to T1 and T2 (but not T1 to T2) in the meditation group, suggesting that the combination of vegan diet and meditation contributed to durable changes in bacterial composition. *Bifidobacteria* and *Lactobacillus* genera are considered probiotics [50], conferring positive health benefits to their host via their metabolic activities. Of interest, a double-blinded placebo-controlled trial conducted in medical students exposed to academic stress demonstrated that daily consumption of *Lactobacillus casei* improved sleep quality [51]. Gut microbial *Lactobacillus* counts were also associated with improved sleep quality in patients with bipolar disorder [52]. In addition, a negative correlation was identified between *Bifidobacterium* counts and cortisol levels in those patients, raising the possibility of a bacterial role in stress response [52].

A cross-sectional study [11] investigated the differences in gut microbial composition in 12 long-term meditators consuming a vegan diet and 12 omnivorous subjects who did not practice mediation. The abundance of 12 taxa in the samples of the meditation group was significantly higher than those in the control group, including *Bifidobacterium* and *Collinsella*.

Vegan diet and its effect on the gut microbiota has been well-studied during the last decade. Recently, a systematic review of the impact of vegan or vegetarian diet on gut microbial composition compared to an omnivorous diet identified no consistent association between a vegan or vegetarian diet and that of omnivores; in fact, some discordant results were noted [53]. At the genus level, significantly lower levels of Bacteroides in vegans were observed in some studies, although the majority of the included studies could not find any differences. Similarly, *Bifidobacterium* and *Lactobacillus* tended to be of lower abundance in vegans, but only in a small number of studies, with the majority not identifying differences between groups [53]. In contrast, a narrative review [54] concluded that compared with omnivores, vegetarians have higher gut microbiome diversity, and higher abundance of *Prevotella, Clostridium, Lactobacillus, Ruminococcus, E. rectale*, and *F. prausnitzii*, but lower abundance of *Bacteroides Bifidobacterium.*

Our findings relating to *Bifidobacterium* and *Lactobacillus* are similar to those identified in the above mentioned systematic review. Bifidobacterium increased at T3 compared to T1 and T2, but not at T2 compared to T1 that represents the period of vegan diet intervention. Higher Bifidobacterium abundance at T3 would suggest the possibility for habitual diet (rather than a vegan diet) to explain this finding, however this remains speculative as dietary data are not available to explore this hypothesis. An alternative hypothesis to explain higher bifidobacterium abundance at T3 could include effects of meditation. In the absence of stool collection immediately post meditation, this hypothesis remains speculative as well.

Although vegan diet quality may be variable, dietary fiber intake increases compared with vegetarians and omnivores [55]. Resistant starch is a form of dietary fiber that escapes small intestinal digestion and is fermented by the gut microbiota. Resistant starch sub-types have been associated with increased abundance of *Bifidobacterium*, *Collinsella,* and *Romboutsia* [56]. While unexpectedly, *Collinesella* and *Romboutsia* decreased in abundance during the strict vegan intervention (T1-T2), an increased abundance of both these genera at T3 compared to T2 and T1 in the intervention group was observed. These genera produce SCFAs which have an important role in intestinal immune cell regulation and maintenance of immune homeostasis, demonstrating an immunomodulatory potential [57]. In our study, no changes in major SCFAs were seen throughout the trial duration. However, changes in overall metabolite profiles and higher proportions of branched SCFA (BCFA) that may serve as markers of protein fermentation [58] were observed among meditators at T2. Although the broader effects of BCFA in human health are not yet well described, a recent report suggested the possibility that iso-butyrate may improve 5-hydroxytryptophan levels through upregulation of *Tph1* expression to yield anti-depressant-like effects [59].

Increasing evidence is mounting for the role of gut bacteria and their metabolites in host-signaling responses along the gut-brain axis. Microbial components may influence brain functions via neuroendocrine and neuroimmune mechanisms [60]. A lower abundance of *Bifidobacterium* and *Lactobacillus* was previously observed in patients with major depressive disorders compared to healthy controls [61]. *Lactobacillus plantarum* DR7 intervention alleviated stress and anxiety in adults, and improved memory, attention, and emotional cognition. In addition, it was previously reported a positive correlation between *Lactobacillus plantarum DR7* administration, gene expression of dopamine B-hydrolase, and abundance of genus *Romboutsia* suggesting a potential microbial impact on neurotransmitter function [60]. We recently demonstrated that in 632 adults who completed the Samyama program (including vegan diet and meditation), had improved scores of depression, anxiety, vitality, resilience, and joyfulness compared to baseline [22].

The body mass index (BMI) is lower in meditators compared to controls, and this may be a result of frequent yoga practices and greater adherence to vegetarian or vegan diets which frequently are observed in tandem. Microbial dysbiosis, specifically the Bacteroidetes to Firmicutes ratio is negatively correlated with BMI, which may be related to increased energy harvest, resulting in weight gain over time [62]. Additionally, a low-fat vegan diet was shown in a previous study to induce significant changes in gut microbiota which were related to changes in weight and body composition in otherwise healthy participants [63]. Therefore, BMI may have contributed mechanistically to our gut microbiome findings.

In this study, we demonstrated differences in several fecal metabolites in meditators over time, with the greatest differences observed at T2 (vegan diet period) compared to the other two timepoints. This suggests that a vegan diet leads to functional changes in the fecal microbiome. Although our current findings are preliminary and further study will be required to identify the affected microbial pathways that may drive altered metabolite profiles, others have demonstrated that gut bacterial metabolic activity differs significantly between those consuming vegan and omnivorous diets [64]. Although between group differences were not significant, the microbial compositional and functional changes observed in meditators but not the controls at T2 are consistent with vegan diet intervention, and findings at T3 could either be related to the effects of Samyama or a return to the usual diet. The authors cannot conclude the basis behind this change based on the data in this study as we do not have adequate dietary data. The strengths of this study include a large meditator sample size, longer follow-ups, and objective data collection that include blood samples, fMRI, and stool sample collection. As described in the introduction, several objective findings that include a sustained reduction in anxiety, depression, improved brain connectivity have been previously published from this study data [22, 25].

Study limitations include unselected study participants at baseline (underlying medical diagnoses are unknown therefore disease related microbial dysbiosis may have influenced study findings), lack of both baseline dietary assessment and validated measure of dietary compliance, and absence of stool collection immediately post intervention to inform the combined effects of vegan diet and meditation (before returning to habitual diet). Furthermore, controls did not have the same dietary restrictions as meditators. This includes restrictions for garlic, onions, peppers, eggplant, asafoetida, coffee, and tea. Additionally, while the T2 microbial composition and functional changes at T2 are likely reflective of vegan diet in the intervention group, impact of the pre-requisite yoga practices on these measures is unknown. Future studies may build from this body of work to rigorously detangle the relationship between diet and yoga practices. It is also important to mention that BMI is significantly lower in the meditator pool and this may have impacted findings. Finally, it was not determined if the controls conducted yoga/meditation practices or what practices were observed by meditators after completing the Samyama program, and if meditators returned to their habitual diet post Samyama.

## Conclusion

This non-randomized controlled longitudinal prospective study is among the first and largest to explore the gut microbiome and metabolome profile after an advanced Samyama meditation program delivered with a vegan diet. First, we observed changes in meditator beta diversity after Samyama. Additionally, after the preparatory phase, there was an increase in branched short-chain fatty acids and changes to other metabolite composition. While the implications of these results are not fully understood, these findings do pave the way for further exploration of the impact of meditation and diet on the gut-brain axis. Further study is warranted to validate current observations, investigate significance and mechanisms of action related to diet, meditation, and microbial composition and function, on psychological processes, including mood.

## Data Availability

The data is available in from the United States National Library of Medicine and the National Center for Biotechnology Information. The BioProject is 904395 (https://www.ncbi.nlm.nih.gov/bioproject/904395).

## Abbreviations

ANOVA: Analysis of variance
ASV: Amplicon sequence variant
BCFA: Branched chain fatty acid
BDNF: Brain-derived neurotropic factor
HPA: Hypothalamus-pituitary-adrenal
PCoA: Principal coordinate analysis
SCFA: Short-chain fatty acid
BMI: Body mass index
